# Growth of ZnO Nanorods on Stainless Steel Wire Using Chemical Vapour Deposition and Their Photocatalytic Activity

**DOI:** 10.1155/2014/252851

**Published:** 2014-01-22

**Authors:** Siti Nor Qurratu Aini Abd Aziz, Swee-Yong Pung, Nurul Najiah Ramli, Zainovia Lockman

**Affiliations:** ^1^Science and Engineering of Nanomaterials Team, School of Materials and Mineral Resources Engineering, Engineering Campus, Universiti Sains Malaysia, 14300 Nibong Tebal, Pulau Pinang, Malaysia; ^2^Green Electronic Nanomaterials Group, School of Materials and Mineral Resources Engineering, Engineering Campus, Universiti Sains Malaysia, 14300 Nibong Tebal, Pulau Pinang, Malaysia

## Abstract

The photodegradation efficiency of ZnO nanoparticles in removal of organic pollutants deteriorates over time as a high percentage of the nanoparticles can be drained away by water during the wastewater treatment. This problem can be solved by growing the ZnO nanorods on stainless steel wire. In this work, ZnO nanorods were successfully grown on stainless steel wire by chemical vapour deposition. The SAED analysis indicates that ZnO nanorod is a single crystal and is preferentially grown in [0001] direction. The deconvoluted O 1s peak at 531.5 eV in XPS analysis is associated with oxygen deficient, revealing that the ZnO nanorods contain many oxygen vacancies. This observation is further supported by the finding of the small *I*_uv_/*I*_vis_ ratio, that is, ~1 in the photoluminescence analysis. The growth of ZnO nanorods on stainless steel wire was governed by vapour-solid mechanism as there were no Fe particles observed at the tips of the nanorods. The photodegradation of Rhodamine B solution by ZnO nanorods followed the first-order kinetics.

## 1. Introduction

Zinc oxide (ZnO) is a semiconductor material with a wide bandgap energy (3.37 eV) and large exciton binding energy (60 meV) at room temperature. Attributed to these unique properties, ZnO nanostructures such as nanorods (NRs) [1], nanofibres (NFs) [2], and nanobelts (NBs) [3] have lately been the focus of research activities. These ZnO nanostructures can be used for applications in light-emitting diodes [4] solar cells [5], nanogenerators [6], gas sensors [7], photodetectors [8], and UV lasers [9]. Different techniques have been developed for growing these ZnO nanostructures, which includes chemical vapour deposition (CVD) [10], physical vapour deposition (PVD) [11], molecular beam epitaxy (MBE) [12], pulsed laser deposition (PLD) [13], sol-gel method [14], and electrochemical deposition [15]. Generally, the low growth temperature in solution route (typically under 100°C) affects the crystalline quality of the ZnO nanostructures. The crystal quality of these nanostructures could be improved by carrying out postannealing under certain conditions [16].

The use of wide bandgap semiconductor photocatalysts such as ZnO [17] and TiO_2_ [18] to degrade organic pollutants is another rapid developing research field. Approximately one million tons of synthetic organic dyes are produced every year for applications in textile, paint, cosmetic, and food industries [19]. A significant amount of these organic dyes end as wastewater and are discharged into rivers and seas. The removal of these effluents is critical for the environmental sustainability. Thus, the degradation of organic pollutants by semiconductor photocatalysts is one of the most promising technologies to reduce the adverse effects. It can be considered as the “green” technology for the treatment of environment pollutants with solar energy. The by-products of this technology such as water and CO_2_ are less harmful to the environment. However, usage of these semiconductor photocatalysts in powder form can cause secondary pollution as they can be easily drained away by water. This also results in the efficiency for degradation of organic pollutants to deteriorate over time. In addition, the semiconductor photocatalysts need to be separated from the reaction medium. This exercise is not only time consuming but also costly. The limitations of using semiconductor photocatalysts in powder form can be resolved by growing them onto a rigid substrate such as stainless steel wire. The stainless steel wire functions as a support to the photocatalyst which reduces the incidence of the photocatalyst being easily washed away by the flowing water during the waste water treatment process.

In this paper, ZnO NRs were grown on stainless steel wire using CVD. By controlling the amount of Zn powder, both nano- and micro-ZnO rods could be synthesized on the stainless steel wire. The growth mechanism of ZnO NRs on stainless steel wires was discussed. The photodegradation efficiency of ZnO NRs on removal of organic dye (RhB) under ultraviolet (UV) irradiation was also investigated.

## 2. Materials and Methods

**Figure 1:**
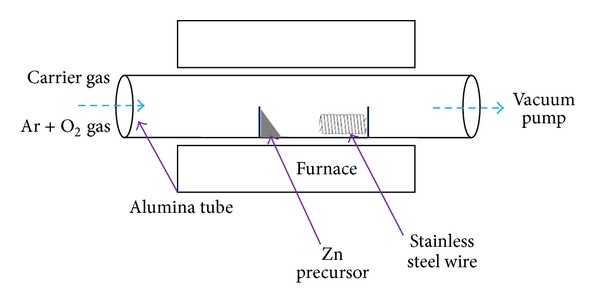
Schematic diagram of CVD system used to grow ZnO NRs.

The stainless steel wire (*ϕ*: 0.05 mm) was used as substrate for the growth of ZnO NRs. The wire was first immersed in acetone and then ultrasonic-cleaned for 10 min. Subsequently, the wire was rinsed with deionized water thoroughly and was left to dry naturally. High purity of Zn powder (99.99%) was used as Zn source. Different amount of Zn powder at 0.3 g, 0.5 g, 1.0 g, 1.5 g, and 2.0 g, respectively, was used to grow the ZnO NRs. As illustrated in Figure 1, the alumina boat was placed in the middle of a horizontal tube furnace. The stainless steel wire was kept at the downstream of the furnace. During the synthesis process, the temperature of the furnace was kept at 650°C with pressure of 1 mbar. A trace amount of oxygen gas (2~3 sccm) was flowed into the furnace for the growth of ZnO NRs. The O_2_ gas was turned off after 30 min and the temperature of the furnace was ramped down to room temperature.

The morphology of the as-synthesized nanostructures was studied by Zeiss Supra 35 VP field emission scanning electron microscope (FESEM) and Tecnai G2 20 S-Twin transmission electron microscope (TEM). The phase and crystallinity of the nanostructures were characterized using Philip PW 1729 X-ray diffractor (Cu K*α*, *λ* = 0.154 nm) (XRD). The photoluminescence property of the nanostructures was characterized by PL Spectrophotometer (Horiba JobinYvon Spectrometer HR 550, 450 W, Xe lamp, 325 nm). The X-ray photoelectron spectroscopy (Omicron Nanotechnology, ELS 5000, MgK*α* radiation) was used to examine the chemical bonds of the sample.

The photocatalytic performance of ZnO was evaluated using RhB dye to represent organic pollutants. The RhB solution with initial concentration of 1 × 10^−5 ^M was prepared using a magnetic stirrer in the dark room. Subsequently, the RhB solution with the ZnO samples was placed together in the reaction cylinder and was irradiated with UV light (360 W lamp, 254 nm) for a period of time. The UV-Vis spectrometer (Cary 50, 553 nm) was used to evaluate the photodegradation of RhB solutions.

## 3. Results and Discussion

### 3.1. Structural and Optical Properties

**Figure 2:**
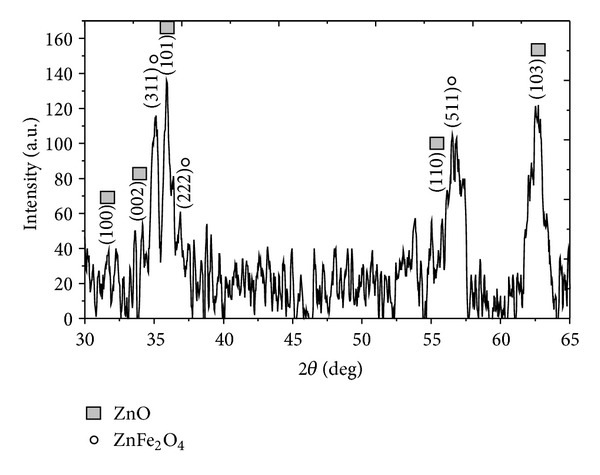
XRD pattern of ZnO NRs grown on stainless steel wire by CVD (650°C, 2 sccm O_2_, and 0.3 g  Zn powder).

The crystal phase of the as-grown sample (650°C, 2 sccm O_2_, and 0.3 g  Zn powder) was analysed by XRD as shown in Figure 2. The diffraction peaks of 31.91°, 34.56°, 36.29°, 56.83°, and 62.94° correspond to (100), (002), (101), (110), and (103) crystal planes, respectively. These diffraction peaks could be indexed as wurtzite ZnO (JSPDS Card no. 01-089-1397) with lattice constants of *a* = *b* = 3.25 Å and *c* = 5.21 Å. In addition, the diffraction peaks at 35.18°, 36.90°, and 56.53° which represent the crystal planes of (310), (222), and (510), respectively, are contributed from ZnFe_2_O_4_. It is possible that Fe was unintentionally doped into ZnO NRs during the synthesis process as the NRs were grown on the stainless steel wire.

**Figure 3:**
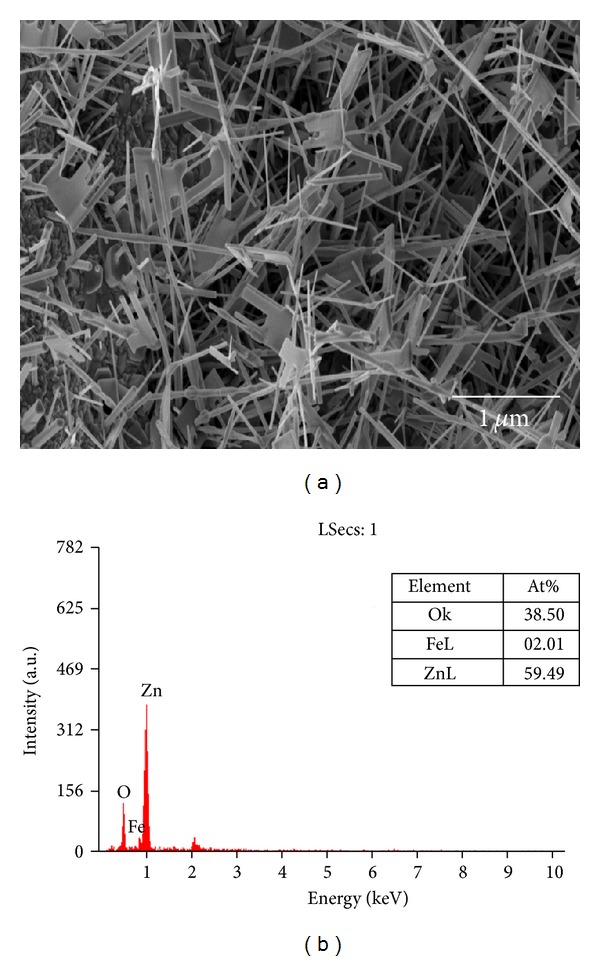
(a) SEM image and (b) EDS analysis of ZnO NRs that are grown on the stainless steel wire using CVD technique (650°C, 2 sccm O_2_, and 0.3 g  Zn powder).

**Figure 4:**
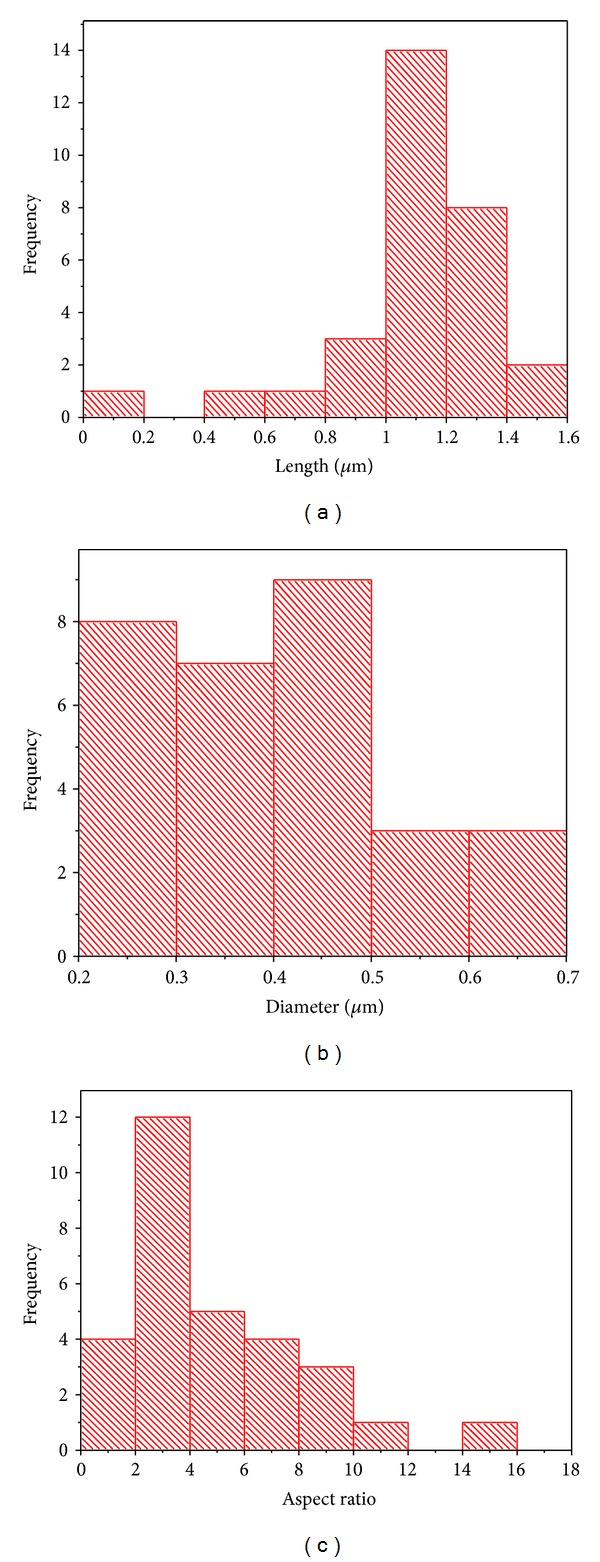
Histogram of ZnO NRs that are grown on stainless steel wire: (a) length, (b) diameter, and (c) aspect ratio (650°C, 2 sccm O_2_, and 0.3 g Zn powder).

As shown in Figure 3(a), ZnO NRs were successfully grown on stainless steel wire using CVD (0.3 g Zn powder and 2 sccm  O_2_). The EDS analysis in Figure 3(b) indicates that the ZnO NRs contain Zn (59.49 At%), O (38.50 At%), and Fe (2.01 At%). The Fe element was contributed from the stainless steel wire.  Figure 4 shows the histograms of length, diameter and aspect ratio of ZnO NRs that are grown on the stainless steel wire. The length, diameter, and aspect ratio of these ZnO NRs are 1.08 ± 0.11 *μ*m, 0.41 ± 0.09 *μ*m, and 2.66 ± 1.09, respectively. The areal density of this ZnO sample is 6.30 ± 0.46 NRs/*μ*m^2^.

**Figure 5:**
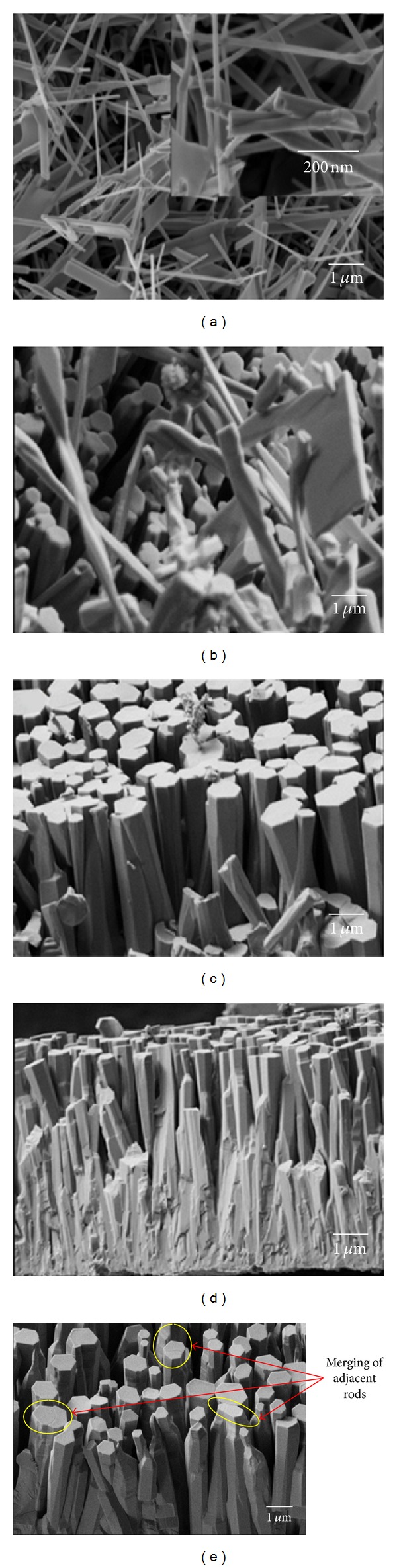
Effect of amount of Zn powder on the growth of ZnO NRs on stainless steel wire: (a) 0.3 g, (b) 0.5 g, (c) 1.0 g, (d) 1.5 g, and (e) 2.0 g, respectively, (650°C and 2 sccm O_2_).

**Figure 6:**
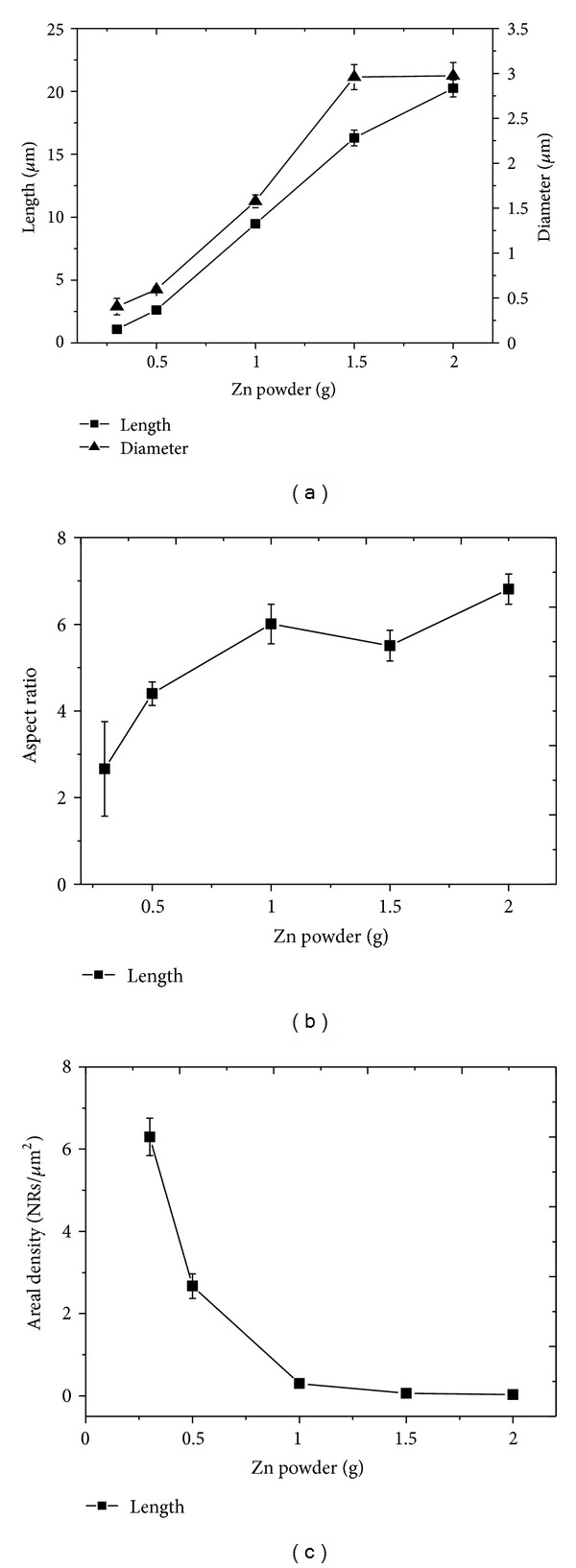
Effect of amount of Zn powder on the growth of ZnO rods: (a) length and diameter, (b) aspect ratio, and (c) areal density.

Figure 5 shows the ZnO NRs grown on stainless steel wire using different amount of Zn powder, that is, 0.3 g, 0.5 g, 1.0 g, 1.5 g, and 2.0 g, respectively. It can be seen in Figure 5 that the ZnO NRs have hexagonal cross sections [002], which is a typical characteristic of ZnO NRs grown in the [0001] direction. As shown in Figure 6(a), the length of the ZnO NRs increases with the increase of Zn powder used in the synthesis, that is, from 1.08 ± 0.11 *μ*m, 2.62 ± 0.10 *μ*m, 9.47 ± 0.32 *μ*m, 16.30 ± 0.63 *μ*m, and 20.24 ± 0.68 *μ*m. The diameter of ZnO NRs also increases from 0.41 ± 0.09 *μ*m, 0.60 ± 0.03 *μ*m, 1.58 ± 0.07 *μ*m, 2.96 ± 0.14 *μ*m, and 2.97 ± 0.15 *μ*m. It was also found that as the amount of Zn powder increased, the ZnO structures grew from nanorods to microrods.

As shown in Figure 6(b), the aspect ratio of ZnO NRs increases from 2.66 ± 1.09, 4.40 ± 0.27, 6.01 ± 0.46, 5.51 ± 0.35, and 6.90 ± 0.35, respectively, with the increase of Zn powder. This is one of the evidence of anisotropic growth of ZnO with the highest growth rate in [0001] direction. However, the areal density of ZnO NRs decreased with the increase of Zn powder as shown in Figure 6(c). The areal density is 6.30 ± 0.45 NRs/*μ*m^2^, 2.67 ± 0.30 NRs/*μ*m^2^, 0.30 ± 0.10 NRs/*μ*m^2^, 0.06 ± 0.04 NRs/*μ*m^2^, and 0.03 ± 0.03 NRs/*μ*m^2^ when the amount of Zn powder used was 0.3 g, 0.5 g, 1.0 g, 1.5 g, and 2.0 g, respectively. This result shows that the NRs were not only grow vertically (length) but also laterally (diameter) with the increase of Zn powder. Some of the adjacent NRs merged together (highlighted in circles in Figure 5(e)) and formed microrods, causing a reduction in areal density. In other words, as the amount of Zn powder increased, more Zn vapour was generated during the synthesis process. This promoted the growth of ZnO from nanorods to microrods with reduction in areal density.

**Figure 7:**
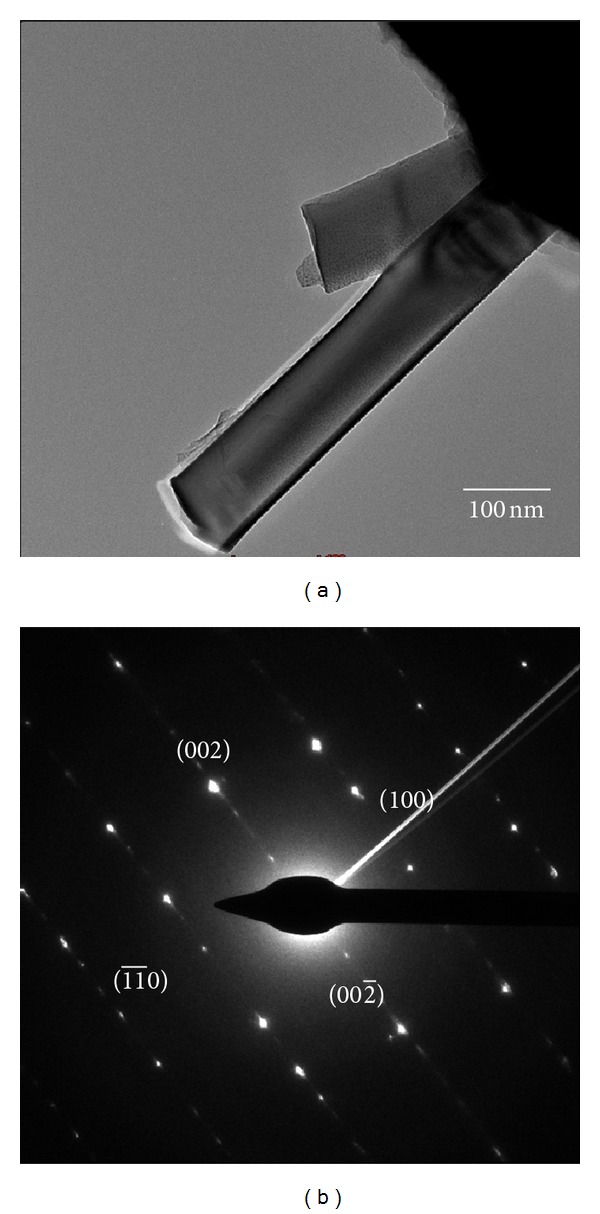
(a) TEM image and (b) SAED image of ZnO NRs grown on the stainless steel wire.

Figure 7(a) shows the TEM image of ZnO NRs with uniform diameter (96 nm) and flat tips. The selected electron area diffraction (SAED) analysis in Figure 7(b) shows that the ZnO NR is single crystal.

**Figure 8:**
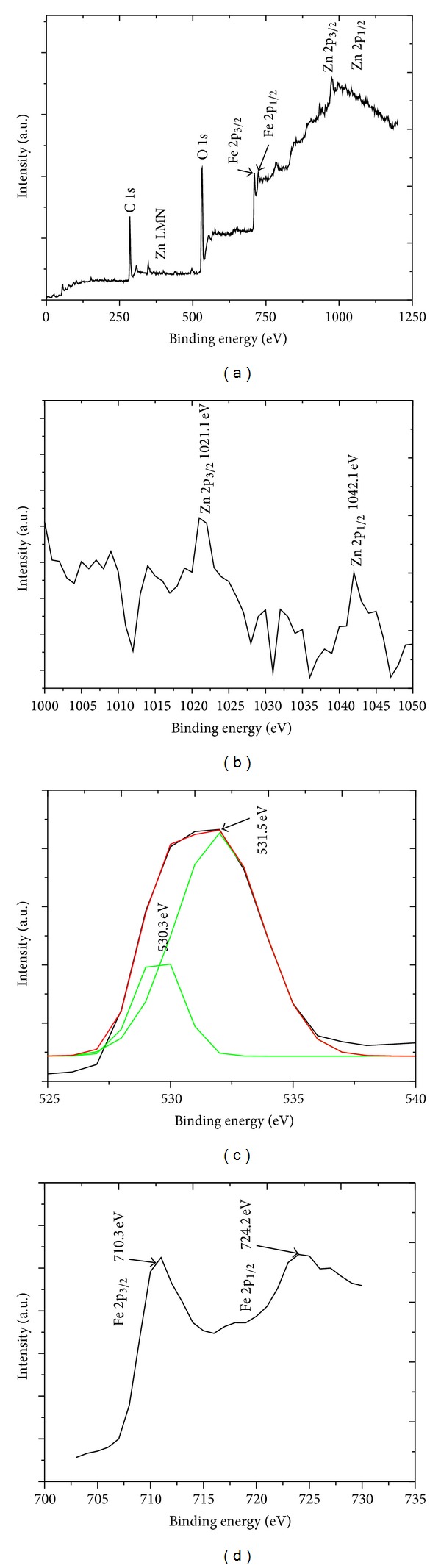
XPS spectra of the ZnO NRs grown on stainless steel wire (a) wide scan, (b) Zn 2p, (c) O 1s and (d) Fe 2p.

Figure 8(a) shows the XPS wide scan analysis of the ZnO NRs grown on stainless steel wire. The binding energies of the spectra have been calibrated by taking the carbon C 1s peak (285 eV) as reference. The carbon peak is due to the unavoidable air exposure during inserting the sample in the XPS chamber. The presence of Zn LMN Auger peak at 497.0 eV and O 1s peak at 532.7 eV indicates that the nanostructures were ZnO as the peaks match well with the references in the Handbook of X-ray photoelectron spectroscopy [20]. In addition, the Zn 2p spectra of the ZnO NRs, which is shown in Figure 8(b), have the binding energies of 1021.1 eV and 1042.1 eV. These peaks can be identified as Zn 2p_3/2_ and Zn 2p_1/2_ lines, respectively. The spin-orbital-splitting energy between the two lines is 21 eV, which is well lying within the standard reference value of Zn (2p) in ZnO [21]. The O 1s peak is prominently identified at approximately 532.7 eV as shown in Figure 8(c). The O 1s XPS is asymmetric, indicating that at least two oxygen species are present in this region. Thus, the peak can be further deconvoluted into two Gaussian peaks, which are located at 530.3 eV and 531.5 eV. The first peak on the low binding energy (530.3 eV) is attributed to the O^2−^ ions on the wurtzite structure of hexagonal Zn^2+^ ion array, which are surrounded by Zn atoms [22]. The second peak (531.5 eV) is associated with oxygen deficient regions within the matrix of ZnO [23].  Figure 8(d) shows that the Fe 2p spectrum comprises two peaks at 710.3 eV and 724.2 eV, which correspond to the Fe 2p_3/2_ and Fe 2p_1/2_, respectively. According to Patil et al. [24], these two peaks are consistent with the bulk *α*-Fe_2_O_3_ peak at 710.8 eV and 724.80 eV. The energy difference between these two peaks is 13.9 eV. This value is the characteristic of Fe^3+^ state, indicating the formation of Fe_2_O_3_. The ZnO NRs were unintentionally doped with Fe from stainless steel wire during the synthesis process.

**Figure 9:**
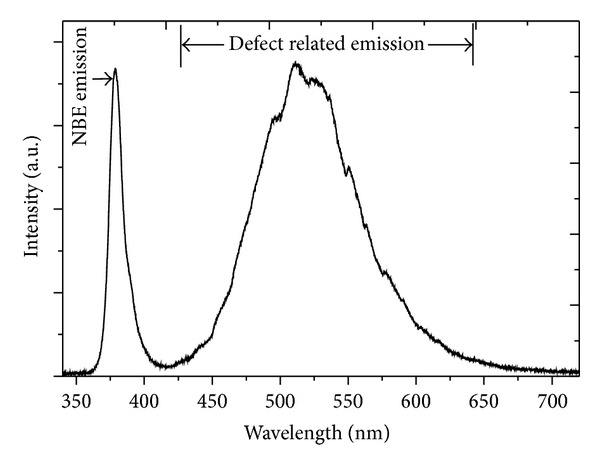
The room temperature PL spectrum of the ZnO NRs grown on stainless steel wire.

A sharp near-band-edge (NBE) UV emission at 379 nm and a broad green emission were observed in the room temperature PL measurement on the ZnO NRs grown on stainless steel wire as shown in Figure 9. The UV peak is attributed to the recombination of free exciton. The green emission peak is generally attributed to the deep level defect in ZnO crystals. According to Shetty and Nanda [25], ZnO contains various defects such as oxygen vacancy (*V*_O_), Zn vacancy (*V*_Zn_), oxygen interstitial (O_*i*_), Zn interstitial (Zn_*i*_), oxygen antisite (O_Zn_), and zinc antisite (Zn_O_). One of the possible origins of this strong green emission is related to the large quantity of the surface oxygen vacancies induced by Fe-related defects of the ZnO NRs. This observation is supported by the XPS analysis shown in Figure 8(c) as the large percentage of O 1s peak is contributed from the oxygen deficiency region (531.5 eV). Since the UV peak represents the NBE emission of ZnO and the green emission is defect-related emission, a good crystal quality of ZnO shall have a large *I*_uv_/*I*_vis_ ratio in PL measurement [26]. Thus, the poor *I*_uv_/*I*_vis_ ratio of PL, that is, ~1, indicates that the ZnO NRs have a relatively poor crystal quality.

### 3.2. Growth Mechanism

**Figure 10:**
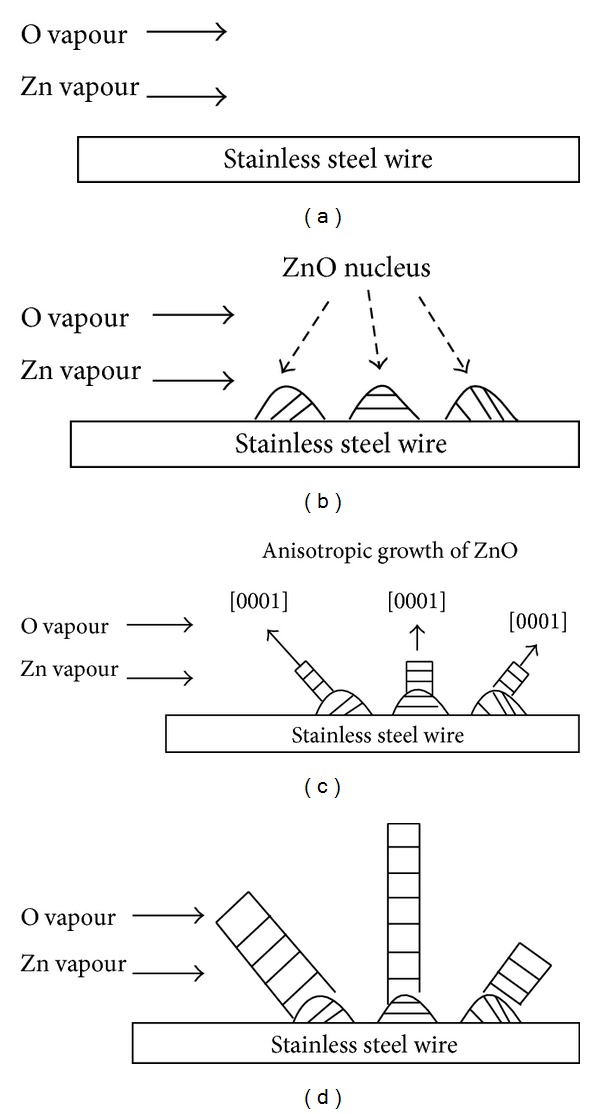
VS growth of ZnO NRs on stainless steel wire: (a) condensation of Zn and O vapours, (b) formation of ZnO nucleus, (c) anisotropic growth of ZnO nucleus, and (d) formation of ZnO NRs on stainless steel wire.

The most common mechanism used to describe the growth of ZnO NRs is vapour-liquid-solid (VLS) mechanism. One of the unique characteristics of nanorods grown under VLS mechanism is the presence of metal alloy nanoparticles at the tips of the nanorods. However, the growth of ZnO NRs on stainless steel wire is concluded to follow vapour-solid (VS) mechanism as no metal particles could be observed at the tips of the NRs (Figure 3(a)). As illustrated in Figures 10(a) and 10(b), zinc and oxygen vapours condense on the surface of stainless steel wire to form ZnO nucleus during the synthesis process. The surface energy of crystal planes of ZnO nucleus contributes to the effectiveness of capturing the subsequent adsorbed Zn and O atoms. This energy also affects the growth rate and the proportion of crystallographic planes of the nanostructures [25]. In the case of ZnO, the (002) plane has the lowest surface free energy, followed by the {100} planes. Thus, ZnO NRs have the highest growth rate in [0001] direction, resulting in anisotropic growth of nanorods (Figure 8(c)). Eventually, ZnO NRs are formed towards the end of the synthesis process (Figure 10(d)).

### 3.3. Photocatalytic Study

**Figure 11:**
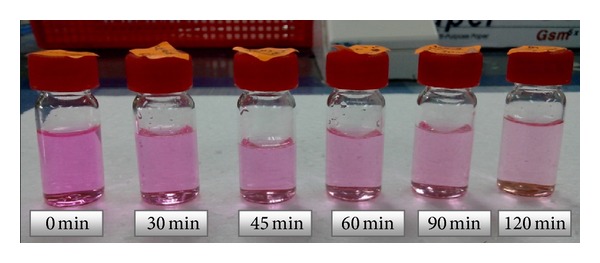
Degradation of RhB solution under UV light irradiation (254 nm) over a period of time by ZnO NRs.

**Figure 12:**
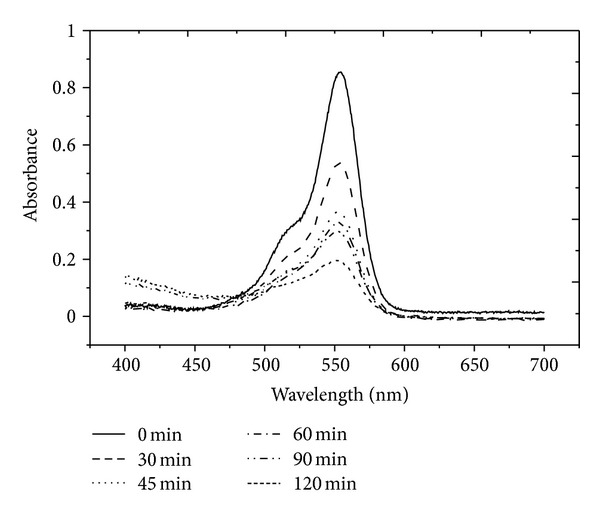
Deterioration of absorption spectra of RhB solutions (553 nm) over a period of UV irradiation time by ZnO NRs.

The effectiveness of ZnO NRs grown on stainless steel wire as photocatalyst to degrade RhB solution under UV light (254 nm) was studied. The ZnO in Figure 11 shows the photodegradation of RhB solution by ZnO NRs as photocatalysts over a period of UV irradiation time. The RhB solution decolourized gradually. The degradation of the RhB solution was monitored by the deterioration of absorbance at 553 nm.  Figure 12 shows the absorbance peak of RhB solution that degraded by ZnO NRs. It decreased from the initial absorbance of 0.853 (a.u.) to 0.537 (a.u.) after 30 min of UV irradiation. After 120 min, the RhB absorbance peak decreased to 0.194 (a.u.).

**Figure 13:**
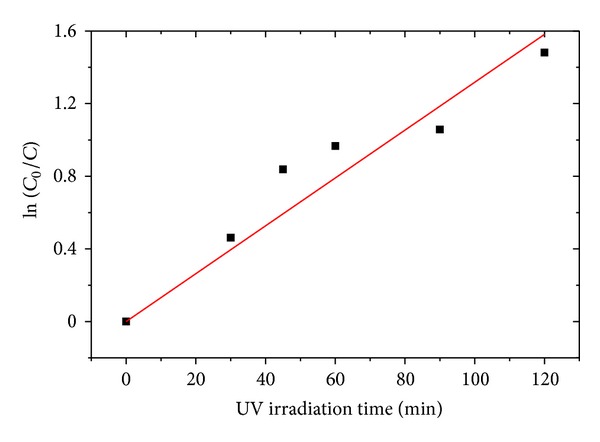
Degradation of RhB aqueous solution by ZnO NRs and ZnO microrods under UV irradiation.

It is known that the absorption of solution (*A*) is proportional to the concentration (*C*) of the absorbing species as stated in Beer-Lambert Law [27]. Thus, the absorbance data is plotted as ln (*C*_0_/*C*) of the time-dependent normalized dye concentrations (which is the ratio between the initial concentration and the concentration upon reaction) as shown in Figure 13. The linear plots in Figure 13 indicate that the degradation of RhB solution by ZnO NRs follows pseudo-first-order kinetics. The rate constant of ZnO NRs is 0.013 ± 0.001 min^−1^.

The above observation can be explained as follows. The electrons and holes are generated in the ZnO upon UV irradiation as the UV light has the energy greater than the bandgap of ZnO. These charge carriers need to travel to the surface of ZnO rods for the formation of free radicals. In this case, oxygen acts as an electron acceptor by forming superoxide radical anions (O_2_^•−^) whereas holes react with water adhering to the surfaces of semiconductor particles to form highly reactive hydroxyl radicals (^•^OH). These free radicals are capable of degrading the RhB molecules into less harmful by-products such as water and acids. As these reactions occur on the surface of ZnO exposed to solution, a larger exposed surface allows a more effective charges transfer for the formation of free radicals.

## 4. Conclusions

ZnO NRs were successfully synthesized on stainless steel wire via CVD. The deconvoluted O1s peak at 531.5 eV in XPS analysis is associated with oxygen deficient defects in ZnO. Thus, ZnO NRs grown on stainless steel wire contained many crystal defects which are mainly related to oxygen vacancies. This observation agrees well with the PL result. The PL spectra of ZnO NRs grown on stainless steel wire show a low *I*_uv_/*I*_vis_ ratio, that is, ~1. This indicates that the ZnO NRs have a relatively poor crystal quality. It is crucial to control the amount of Zn powder used in the synthesis process as it promotes the growth of ZnO from nanorods to microrods. The reduction in areal density of ZnO rods was attributed to the merging of adjacent rods. The growth of ZnO NRs on stainless steel wire was governed by VS mechanism as no foreign catalyst could be observed at the tips of NRs. The photodegradation of RhB solution by the ZnO NRs grown on stainless steel wire under UV irradiation followed the first-order kinetics with rate constant of 0.013 ± 0.001 min^−1^.
